# Disclosure or non-disclosure? Misdirection and secrecy around communicating with schools about the diagnosis of anorexia nervosa

**DOI:** 10.1186/2050-2974-1-S1-P9

**Published:** 2013-11-14

**Authors:** Evelyn Bowtell, Julie Green, Rosalie Aroni, Susan Sawyer

**Affiliations:** 1 Centre for Adolescent Health, Royal Children's Hospital, Department of Paediatrics, The University of Melbourne, Murdoch Childrens Research Institute, Australia; 2 Department of Paediatrics, The University of Melbourne, Murdoch Childrens Research Institute Royal Children's Hospital Education Institute, Parenting Research Centre, Australia; 3 School of Public Health and Preventive Medicine, Faculty of Medicine, Nursing and Health Sciences, Monash University, Australia

## 

The aim of this qualitative study was to improve the understanding of the interface between health and educational sectors. Parents of adolescents with anorexia nervosa (AN, n=11), cancer (n=11) and cystic fibrosis (CF, n=16) were recruited through two tertiary hospitals in Victoria. Audio-recorded in-depth interviews were conducted and transcribed verbatim. Consistent themes emerged across the three cohorts, with parents agreeing that schools needed to know of a diagnosis for health safety, academic support and positive peer relationships. However there were significant condition-specific differences. Only parents of adolescents with AN engaged in misdirection (providing a different diagnostic label) or secrecy; a significant number elected not to disclose the AN diagnosis to schools in response to their child's request. In contrast, parents of adolescents with CF who reported that their children did not wish schools to know of the diagnosis when secondary school commenced overruled such requests. Diagnosis disclosure did not emerge as an issue for adolescents with cancer; all parents promptly informed the school of the diagnosis. In summary, parents articulated consistent reasons across disease cohorts for sharing the diagnosis of a chronic health condition with schools, yet experienced different challenges in implementing this, with implications around health safety, academic support and peer relationships.

